# Full Genome Sequence of a Reassortant Human G9P[4] Rotavirus Strain

**DOI:** 10.1128/genomeA.01284-14

**Published:** 2014-12-11

**Authors:** Jamie Lewis, Sunando Roy, Mathew D. Esona, Slavica Mijatovic-Rustempasic, Christine Hardy, Yuhuan Wang, Margaret Cortese, Michael D. Bowen

**Affiliations:** a Division of Viral Diseases, National Center for Immunization and Respiratory Diseases, Centers for Disease Control and Prevention, Atlanta, Georgia, USA; b Long Beach Memorial Medical Center, Long Beach, California, USA

## Abstract

This is a report of the complete genomic sequence of a reassortant rotavirus group A G9-P[4]-I2-R2-C2-M2-A2-N2-T2-E6-H2 strain designated RVA/Human-wt/USA/ LB1562/2010/G9P[4].

## GENOME ANNOUNCEMENT

The genome of group A rotaviruses (RVA) is composed of 11 segments of double-stranded RNA (dsRNA) and encodes 6 structural proteins (VPs) and 5 or 6 nonstructural proteins (NSPs): VP7, VP4, VP6, VP1 to -3, and NSP1 to -5/6 (1). The 11 genes are classified using the convention Gx-P[x]-Ix-Rx-Cx-Mx-Ax-Nx-Tx-Ex-Hx, where x indicates the genotype number (2). Most human RVA strains possess either the Wa-like constellation (I1-R1-C1-M1-A1-N1-T1-E1-H1) or the DS-1-like constellation (I2-R2-C2-M2-A2-N2-T2-E2-H2), which are believed to have evolved from pig and cow RVAs, respectively (3).

Here, we report the full genome sequence of RVA strain RVA/Human-wt/USA/LB1562/2010/G9P[4] (abbreviated LB1562), detected in a stool sample collected through the National Rotavirus Strain Surveillance System (4) from a 4-year-old child who was treated at the Long Beach Memorial Medical Center in Long Beach, CA, in 2010. RVA dsRNA was extracted from stool using Trizol reagent (Life Technologies, Grand Island, NY). The sequencing templates were prepared by using sequence-independent whole-genome reverse transcription-PCR (RT-PCR) amplification (5) with slight modifications. PCR amplicons were sequenced by the Illumina Miseq 150 paired-end method by the Genomics Lab, HudsonAlpha Institute for Biotechnology (Huntsville, AL). Illumina sequence reads were analyzed using CLC Genomics Workbench 6.0. A combination of *de novo* assembly followed by mapping to a G9P[4] reference strain was used to obtain the full-length genome of strain LB1562. The sizes of full-length segments 1 to 11 are 3,302, 2,687, 2,591, 2,359, 1,566, 1,359, 1,066, 1,059, 1,061, 750, and 816 bp and the open reading frames (ORFs) for these segments are 3,265, 2,641, 2,506, 2,326, 1,459, 1,192, 940, 952, 979, 526, and 601 bp, respectively. Genotype assignment for each gene was accomplished using Blast (http://blast.ncbi.nlm.nih.gov/Blast.cgi) and RotaC 2.0 (http://rotac.regatools.be/).

The full genotype constellation for strain LB1562 is G9-P[4]-I2-R2-C2-M2-A2-N2-T2-E6-H2. Nine genes (VP1, VP2, VP3, VP4, VP6, NSP1, NSP2, NSP3, and NSP5) were highly similar to DS-1-like human RVAs. The LB1562 VP1 gene is closely related to wild-type human G9P[4] strains, the reference DS-1 strain, and a caprine strain, G034 (GenBank accession no. GU937877). The LB1562 VP4 gene is similar to G2-associated P[4] genes from Australia and Bangladesh (97.4% to 99.6% identity) and shares 99.7% to 100% identity with VP4 gene sequences of G9P[4] strains from Guatemala, Honduras, and Mexico (6). The LB1562 VP6 gene shares 98.8% to 99.2% identity with wild-type G9P[4] strains and 99.7% identity with VP6 gene sequences of G9P[4] strains from Latin America (6).

The LB1562 VP7 gene was most similar to G9 lineage III genes (7). It shares 99.5% to 99.6% identity with other lineage III strains and displays 99.9% to 100% identity with VP7 gene sequences of G9P[4] strains from Guatemala, Honduras, and Mexico (6). The LB1562 NSP4 gene was 99.6% to 100% identical to genotype E6 NSP4 sequences of G9P[4] strains from Latin America (6). This is the first complete RVA sequence with genotype G9-P[4]-I2-R2-C2-M2-A2-N2-T2-E6-H2.

### Nucleotide sequence accession numbers.

The strain RVA/Human-wt/USA/LB1562/2010/G9P[4] sequences have been deposited in GenBank under accession numbers KC782514 to KC782524.
